# Hospitals Should Offer Straws Only on Demand to the Public and Patients—An Environmental and Patient Care Imperative

**DOI:** 10.3390/ijerph21020127

**Published:** 2024-01-24

**Authors:** Deepak Agrawal, Armin Bashashati

**Affiliations:** 1 Department of Medicine, Dell Medical School, Dell Seton Medical Center, University of Texas at Austin, Austin, TX 78712, USA; 2 College of Natural Sciences, University of Texas at Austin, Austin, TX 78712, USA

**Keywords:** straws, healthcare greenhouse gases, carbon footprint, healthcare emissions

## Abstract

Plastic straws have become the poster child of waste associated with unnecessary single-use plastics. The visibility of straws littering the land and marine environments has influenced proposals to decrease the use of plastic straws. These include bans on plastic straws at the city, state, and national levels and by many corporations. However, most hospitals continue to use plastic straws in hospital dining areas and for patients. Hospital dining areas are like other public dining areas, so eliminating plastic straws should be straightforward. Regarding the use of straws by patients, we review evidence and propose that patients should not be routinely given straws. Instead, physicians and nursing staff should request straws depending on the patient’s medical needs. Plastic straws make up only a tiny fraction of hospital waste but can be a catalyst to create awareness and decrease unnecessary plastic use in other areas of the hospital. Mitigating climate change requires a concerted effort from hospital leadership and healthcare providers. Only then will hospitals be able to fulfill their climate pledge of net zero emissions by 2050.

## 1. Introduction

Plastic straws have become the poster child of wasteful single-use plastics in efforts to decrease carbon emissions and climate change. Most of the estimated 175–500 million plastic straws used daily in the United States are a product of habit, convenience, and mostly unnecessary [1,2]. A plastic straw weighs 0.4 g, equivalent to using 70,000 tons of plastic annually. Lifecycle analyses suggest that a single straw produces 1.5–5.8 g of carbon dioxide equivalent in a year, which, for all the plastic straws used in the US, would be equivalent to 75,000 gas cars driven for one year [3].

Aside from emissions, plastic straws litter the terrestrial and aquatic environment and are a significant source of microplastics, which have infiltrated the food chain and are associated with many illnesses and cancers. Most of this was known, but not much was done until a viral, graphic video of a plastic straw being pulled out from the nostril of an endangered turtle generated a visceral, emotional response and a movement to eliminate plastic straws [4]. An estimated 8 million tons of plastic waste enter the ocean every year, and it is projected that the ocean will have more plastic than fish by 2050 [5]. Plastic pollution also adversely affects soil health, reducing agricultural yields [6].

Many governments and organizations have eliminated plastic straws due to public pressure and to promote environmental health. Prominent corporations include Starbucks, Disney, American Airlines, Marriott, and Hyatt, while a few cities are Seattle, New York, and Berkeley. Medical facilities are generally exempted from these bans [7]. But should they be? Hospital straws are used in dining areas and in patient food trays. The hospital dining areas and cafeterias are used by visitors and hospital staff, which are like public dining areas, where the use of straws has been restricted by some cities and organizations. While a few patients may benefit from the use of straws, most patients do not need them. The number of straws used in hospitals is significant. For example, almost one million straws are used annually by the Johns Hopkins Hospital [8], three million straws annually by five hospitals of the Inova Health System [9], and fifteen million by the National Health Services in England [10]. The hospitals bear as much responsibility, if not more, as cities and corporations to mitigate the effects of climate change. Healthcare systems are in the business of treating and preserving the population’s health, but they adversely affect the population’s health from their high carbon footprint.

Worldwide, the healthcare sector is responsible for an estimated 4.6 percent of total greenhouse gas emissions (GHG) [11]. In the United States, the share is 8.5 percent [12]. This has not gone unnoticed. In 2022, 102 organizations representing 837 hospitals signed the voluntary “White House Health Sector Climate Pledge”, agreeing to cut greenhouse gas emissions by 50% by 2030 and achieve net zero emissions by 2050 [13]. The editors of major medical journals recently declared, “overall environmental crisis is now so severe as to be a global health emergency” [14]. Responding to this emergency would require implementing every possible opportunity to reduce waste, and this should include disposable plastics such as straws.

Some healthcare systems have addressed the use of straws in hospitals. National Health Services (NHS) United Kingdom, which has a similarly ambitious target of net zero emissions by 2045, targeted the use of plastics in hospital canteens. In 2018, the NHS purchased over 200 million single-use plastic catering items, including 15 million plastic straws. In 2019, retailers operating in hospitals committed to cutting avoidable plastics, starting with straws and stirrers and expanding to cutlery, plates, and cups over the next twelve months [10]. The NHS took this step even before plastic straws were banned in England in 2020. In the US, Inova Health System, a 5-hospital system in Northern Virginia; Dignity Health, a 39-hospital system in the Western United States; Intermountain Healthcare; and Johns Hopkins Hospital have eliminated plastic straws and give biodegradable straws when requested. The elimination of plastic straws from these hospitals is mostly from public areas. However, straws are still included in patients’ food trays whether they want it or not and whether they need it. 

In this review we address the questions: Should straws be given to all patients or only as needed? Does the routine use of straws benefit patients? Should hospital dining areas freely offer straws or only on request? Should plastic straws be replaced by biodegradable straws? And, what is the carbon footprint of straws in the hospital? We hope that the data presented and arguments will help hospitals implement a straw policy that is evidence-based, patient-centric, and sustainable.

## 2. Materials and Methods

Three searches were performed. First, we performed a content analysis where key publications on the use of straws for swallowing were identified with PubMed and Google Scholar searches using the keywords “straws” and dysphagia, aspiration, pneumonia, swallowing, drinking, and cups. The outputs of these searches were combined, and duplicates were removed. All abstracts in English were reviewed to select relevant articles. When abstracts were not available, full articles were reviewed. The articles were selected if they included data on the ease of swallowing and the risk of aspiration in adults when using straws and were published between the years 1990 and the present. Pertinent references from each selected article were also reviewed.

The second PubMed and Google Scholar search used the keywords straws and lifecycle analysis and sustainability. The inclusion criteria included articles with lifecycle analysis performed using data from the United States. The third search was a web search for history, evolution, and regulations of the use of plastic straws.

## 3. History of Straws

A brief history of straws clarifies how they have evolved and why we continue using them. The first descriptions of devices resembling straws were 5000–7000 years ago when Sumerians of Mesopotamia drank beer by placing long, hollow sticks into large vats that were too heavy to lift and to avoid the solid byproducts of fermentation [15,16,17]. The straws were mostly made of precious metals and hence restricted to the elite. Mass-produced straws made from ryegrass came to market in the 1800s, but these straws often disintegrated quickly and gave the drink a musty, unpleasant flavor [17,18]. Marvin Stone, a cigarette roller manufacturer, designed a paper straw by wrapping paper strips around a pencil and then removing the pencil. He patented his design in 1888 and continued to innovate, for example, applying a thin layer of wax to the paper to increase the durability and avoid dissolution in liquids [18]. The straws were marketed as tools of “health, convenience and economy” while also making them a status symbol. In a trade ad, Stone remarked, “All first-class clubs, hotels, saloons, restaurants, etc., use my straws” [19]. The paper straw quickly became popular, with daily production reaching two million at its peak.

In the 1930s, Joseph Friedman made the straw bendable by inserting a screw into the straw, wrapping floss around the screw’s grooves, and then taking out the screw. He patented his invention and, in 1939, founded Flex-Straw Company (Los Angeles, USA) [17]. His first sale was to a hospital rather than a restaurant when hospital staff realized patients could drink liquids while lying in bed. An advertisement by FlexStraw promoting straws in hospitals commented on “maximum comfort and efficiency” and even pictured a patient drinking with a straw while lying flat [20]. The ad also highlighted “saving valuable time at current high labor costs.” The straws have been part of patients’ food trays since, with the clinical utility not questioned.

By the 1960s, paper straws were replaced with plastic straws. This was an opportune time for the straw companies with the fast-growing popularity of fast food and drinks on the go. So, the use of straws evolved from an ingenious invention to a product of style, convenience, and habit. Globally, one billion straws are used daily [21], and every plastic straw ever made, unless burnt, still exists today.

## 4. Studies Evaluating Straws as a Swallowing Aid

A discussion about the utility of straws for patients requires that all stakeholders (patients, healthcare providers, hospital administrators, and the general population) understand the available evidence. Using straws in hospitals has persisted under the perception that straws make drinking liquids easier and safer for hospitalized patients. The safety of drinking with a straw or a cup is generally determined by assessing the risk of aspiration using a Penetration–Aspiration Scale (PAS), which uses X-rays or endoscopy to determine how much drinking liquid enters the airways and the ability of the patient to clear the material. Penetration is defined as the invasion of ingested material into the airway to a level above the vocal folds, and aspiration, a more severe outcome, refers to airway invasion of material below the vocal folds into the trachea (windpipe).

Veiga et al. compared the swallowing of 100 mL of water in 30 elderly patients (>60 years) using a cup or a straw. The cup provided a higher final volume in a shorter time for intake, but there was more liquid spillage. There was no difference in penetration or aspiration between the two groups. In a similar prospective study [22], Butler et al. assessed the swallowing of different liquids and volumes with a cup or straw in fourteen healthy adults >70 years old and found no difference in penetration or aspiration [23]. Daniels et al. studied sequential swallowing with a straw in young and elderly patients and found that the elderly may be at a greater risk of airway compromise due to bolus dropping too low in the pharynx before the onset of the swallow [24].

While the above studies evaluated healthy, elderly subjects, there are a few studies on patients with symptoms of dysphagia (difficulty swallowing). In a prospective, randomized study, 25 patients with symptoms of mild dysphagia were given different volumes of thin liquids to swallow with a cup or a straw, followed by an endoscopic examination. There was no difference in PAS between the cup and straw groups [25]. In another study, 85 poststroke patients underwent X-ray contrast studies using a cup and a straw. No difference in aspiration was noted between the two groups. Notably, some patients showed penetration–aspiration in both the cup-only and straw-only groups [26]. Martin et al. studied breathing patterns in 13 young, healthy patients and found that there is usually a brief period of apnea (not breathing) during swallowing. When taking large, multiple swallows using a straw, this non-breathing time is prolonged and can lead to aspiration [27].

The heterogeneity in studies in the volume of liquid offered, intake instructions, tests performed for evaluation of swallowing, and small sample sizes makes it difficult to make definite conclusions. However, the data suggest no differences in swallowing when using a cup or straw. The choice of a cup or a straw should be individualized after observation and evaluation by an expert, and the indiscriminate use of straw may even be detrimental to some patients. It is accepted that some patients with movement disorders such as benign tremors, Parkinson’s, or other disabilities benefit from the use of straws.

Another downside to using straws is that they encourage improper patient position during meals. One of the marketing ploys for the “flex straw” in the 1940s was to promote the comfort and efficiency of drinking while lying in bed. Unfortunately, hospitals embraced the message, and decades later, most patients continue having meals while reclining in bed. If a patient can get out of bed, then meals should be eaten upright in a chair with feet on a firm surface. This is rarely followed. Brown et al. expressed concern about the underrecognized epidemic of low mobility during hospitalization of older adults and reported that patients who could walk two weeks before admission spent 83% of the hospital stay lying in bed [28]. This can change if the patients are encouraged to get out of bed and have their meals and drinks without straws, as they do at home. Munsterman et al. studied 155 hospitalized elderly patients who ate meals in bed or sitting upright in a chair. There was no difference in aspiration risk and no injuries during transition from bed to chair—however, patients who sat in chairs consumed more food and had higher satisfaction. At discharge, only 11% of patients preferred eating in bed [29].

A quality improvement project to decrease hospital-acquired pneumonia at a general hospital in England was based on compliance with raising bedheads to 30°, sitting patients out of bed for meals, discouraging drinking with straws, and performing regular mouth care. Over six months, there was a 21% increase in the use of adult feeding cups rather than straws and a 26% rise in patients sitting out of bed for meals [30].

## 5. Environmental Impact of Straws

The environmental impact of using straws in the hospital can be extrapolated from studies on overall straw use since the overall impact is likely similar. Straws discarded in the hospital are considered regular (non-biohazard) waste and, thus, ultimately deposited in landfills or incinerated with other waste. The environmental impacts of plastic straws are calculated by performing a Life Cycle Analysis (LCA) across the entire life of the straw (from production to transport to use and disposal).

The two questions are as follows: What is the environmental impact of a plastic straw, and how does it compare to the environmental effects of alternatives to plastic? These alternatives include paper, Polylactic Acid (PLA) made from plant material such as corn starch, seaweed, or sugar cane, and Polyhydroxyalkanoate (PHA) derived from the fermentation of canola oil, jute, and bamboo.

The environmental impacts are generally reported as Global Warming Potential (GWP) for simplicity and easy comparison. The GWP of one plastic straw ranges from 1.46–5.82 g CO_2_ eq, with most studies reporting around 2–3 g CO_2_ eq. The GWPs for PLA, paper, and bamboo in different studies were 5.4–7.15, 1.38–2.6, and 38 g CO_2_ eq, respectively [21,31,32,33,34]. Using the estimate that one million straws are used per 1000 hospital beds [8], and there are 917,000 beds in all US hospitals, the carbon footprint of straws used in US hospitals would be 2–3 billion g CO_2_ eq annually (equivalent to 300,000 gallons of gasoline).

Some studies have attempted to include other environmental impact categories, such as ozone depletion, freshwater, terrestrial and human toxicity potential, and acidification potential, etc. Gao et al. calculated a composite Relative Impact Index (REI) by combining eight impact categories as a single score for a more comprehensive direct comparison between plastic, PLA, and paper straws. The REI values were 2.4 for plastic straws, 6.4 for PLA straws, and 5.1 for paper straws, assuming all the straws were disposed of in a landfill [31]. The reason for the greater environmental impacts of PLA and paper straws was primarily due to their higher total emissions in manufacturing. PLA and paper straws also have higher transportation costs since they are heavier (estimated weights of plastic straw—0.52 g, PLA straw—0.81 g, paper straw—1.15 g) and are often transported from farther locations than paper straws. These higher upstream emissions are insufficient to offset the decrease in end-of-life emissions for biodegradable materials. Notably, biodegradable straws made of materials such as PLA can only be biologically degraded under industrial composting conditions, which rarely happens. A PLA straw discarded in a landfill may thus be environmentally as bad as a plastic straw. We did not review the literature on metal straws since they are reusable and not conducive to use in a hospital setting.

Overall, the environmental footprint of plastic straws is less compared to alternative materials, although a few studies show that paper straws are better than plastic straws [21,34]. A more important conclusion from these studies is that all single-use, disposable straws, whether from plastic or other materials, harm the environment. Further, all single-use alternatives are more expensive than plastic straws. The hospitals are already under financial pressure, so just replacing plastic straws with biodegradable alternatives is unlikely to be implemented by the hospitals. Therefore, the most effective method of combating adverse environmental effects and controlling costs is to reduce the widespread use of straws. The waste hierarchy of “reduce, reuse, recycle” emphasizes “reduce”, and most people, including patients, can indeed consume liquids without straws. However, if a straw is needed, a biodegradable straw may be preferable over a plastic straw.

The results of LCAs should be interpreted in the context of assumptions used in constructing LCAs, such as the source of raw materials, distance and type of transportation, method of disposal, and system boundaries. There are also inherent limitations of LCAs. First, comparative LCAs of plastic straws with other materials assume the same usage. The hope and goal are that replacing plastic with environmentally friendly materials will create awareness and decrease the overall usage of straws. Second, the LCA generally assumes a time horizon of 100 years. Plastics take hundreds of years to decompose; thus, carbon remains trapped in plastic. Hence, the carbon emissions from plastics over the LCA time frame will be lower than those of biodegradable materials such as paper, which decompose and release carbon much faster. Third, a traditional LCA does not measure littering, especially marine litter, and its threat to marine biodiversity, which is one of the biggest problems with plastic pollution. The plastic straws in the hospital are likely disposed of properly and are less likely to cause marine litter. However, the United States also exports its waste to under-resourced countries, which often have lax environmental rules, and the waste generated in the hospitals may end up infiltrating the natural environment. In 2018, the United States produced 292.4 million tons of municipal solid waste and sent 1.07 million tons to lower-income countries (e.g., Vietnam, Malaysia, Thailand, and China) [35].

## 6. Consumers’ Choices and Demands

Changes in practice at hospitals, such as straws available only upon request or need, should consider consumers’ choices, expectations, and demands. Consumer behavior is influenced by education and reasoning, and hospitals can learn from similar policies implemented in other places.

The regulations (bans) on plastic straws by some cities and corporations have faced expected skepticism, resistance, and ambivalence but, by and large, have been accepted by consumers. Wagner & Toews surveyed 133 businesses in San Luis Obispo, California, where an ordinance of “straw only upon request” was passed in 2018 [36]. Most businesses reported no impact on their business; some indicated a slight decrease in costs. The average straw consumption decreased by 32%. For restaurants not using a self-service straw dispenser, the average decrease was 41%. The main concern with bans on straws has been about the accessibility of straws to patients with disabilities. However, this is not an issue when straws are available on request.

Consumers are becoming more conscious about sustainability and their consumption choices. Surveys show that more than 75% of US consumers say a sustainable lifestyle is important and would be open to paying more for sustainable products [37,38]. The studies suggest that when patients, healthcare providers, and visitors are provided with relevant education and rationale, the proposed changes in using straws will be accepted and welcomed.

## 7. Recommendation

### 7.1. Straws in Hospital Dining Areas

Hospital cafeterias and dining halls should give straws “only upon request” (not using a self-service straw dispenser). The plastic straws may be replaced with biodegradable options, preferably those that do not have specific disposal requirements. The catering and hospital staff should be advised, educated, and encouraged about the change. Sharing information that there is precedence in eliminating plastic straws from hospitals will help allay anxiety. For example, Dignity Health, a large healthcare system in the US, eliminated plastic straws and stirrers and reduced plastic use by 11,000 pieces a day [39]. Johns Hopkins Hospital, which purchased almost a million straws yearly, also removed plastic straws [8]. These hospitals replaced plastic straws with paper straws. Our recommendation goes further and suggests giving biodegradable straws “only upon request”.

### 7.2. Straws for Patients

Patients should be given straws based on assessment of need and benefit. If patients are used to drinking from straws, they should be offered straws. Other patients should be assessed for risk of aspiration—for example, patients with disabilities, coughing while eating or drinking, history of aspiration pneumonia or stroke, movement disorders such as benign tremors and Parkinson’s, and poor performance status. Straws are not an automatic default for these patients since some patients may need thickened liquids or swallowing exercises. Drinking with straws may be more harmful in some of these patients. Proper patient position should be emphasized, including getting out of bed while eating.

Speech and swallowing specialists are best positioned to advise about using straws for a patient. Patient and provider education materials should be written and made available. For example, Solent NHS Trust, which provides mental health and learning disability services in certain areas in the UK, in their education document cautions against the indiscriminate use of straws [40].

If straws are recommended, they should satisfy the requirements of individual patients–for example, straws should be durable, resilient enough to withstand jaw closure without breaking, soft, and flexible so as not to damage the teeth of people who bite to stabilize the jaw or who have a bite reflex, suitable for both hot and cold drinks, flexible for angling to the mouth and easily cleaned (if reusable) [41].

## 8. Conclusions

Climate change is now considered a crisis requiring emergency response, which means it is imperative to do everything possible to decrease the carbon footprint of our actions. Using straws in hospitals’ dining areas and for patients is a remnant of habits and promotions when the environment was not a consideration. Patient care protocols rely on evidence, and we present evidence that the indiscriminate use of straws is unnecessary, contributes to environmental pollution, and can even negatively affect patient care. Plastic straws are only a tiny fraction of the problem of plastic pollution and implementing a “straws on request” policy in hospitals will not significantly impact the overall healthcare carbon footprint. However, it would be a worthy first step towards a fundamental shift away from unnecessary single-use plastics in hospitals and a change in mindset towards a greener and sustainable future. Only then can hospitals truly say “Primum non nocere“ (First, do no harm).

## Data Availability

No new data were created or analyzed in this study. Data sharing is not applicable to this article.
